# Rituximab for Children With Difficult-to-Treat Nephrotic Syndrome: Its Effects on Disease Progression and Growth

**DOI:** 10.3389/fped.2019.00313

**Published:** 2019-07-30

**Authors:** Rezan Topaloğlu, Bora Gülhan, Kübra Çelegen, Mihriban İnözü, Mutlu Hayran, Ali Düzova, Fatih Ozaltin

**Affiliations:** ^1^Division of Pediatric Nephrology, Department of Pediatrics, School of Medicine, Hacettepe University, Ankara, Turkey; ^2^Department of Preventive Oncology, School of Medicine, Hacettepe University, Ankara, Turkey; ^3^Nephrogenetics Laboratory, School of Medicine, Hacettepe University, Ankara, Turkey; ^4^Center for Biobanking and Genomics, School of Medicine, Hacettepe University, Ankara, Turkey

**Keywords:** rituximab, nephrotic syndrome, steroid, cyclosporine, growth

## Abstract

**Background:** Since the early 2000s rituximab (RTX) has been thought of as an alternative treatment for steroid-sensitive nephrotic syndrome (SSNS) and steroid-resistant nephrotic syndrome (SRNS).

**Objective:** This study aimed to determine the effects of RTX treatment on disease outcome and growth in pediatric SSNS and SRNS patients.

**Materials and Methods:** The medical records of pediatric SSNS and SRNS patients that began RTX treatment at the mean age of 10.8 ± 5.1 years between 2009 and 2017 were retrospectively reviewed. Additionally, the effect of RTX on growth was evaluated based on patient height, weight, and BMI z scores.

**Results:** The study included 41 children, of which 21 had SSNS and 20 had SRNS. Mean age at diagnosis of NS was 5.8 ± 4.7 years. Mean duration of post-RTX treatment follow-up was 2.3 ± 1.6 years. Among the SSNS patients, 6 and 11 patients were steroid free and calcineurin inhibitor free at the last follow-up visit, respectively. The 1-year cumulative steroid and calcineurin inhibitor doses both decreased after RTX treatment, as compared to before RTX (*P* = 0.001 and *P* = 0.015, respectively). The median height *z*-score at the time of RTX initiation was −1.2 and the median height *z*-score at the last follow-up visit was −0.6 (*P* = 0.044). The median BMI *z*-score decreased from 1.6 (IQR; 0.9–3.0) at the time RTX was initiated to 1.1 IQR; [(−0.7)−2.5] at the last follow-up visit (*P* = 0.007). At the last follow-up visit 4 SRNS patients had complete remission and 4 had partial remission. The 1-year cumulative steroid dosage in the SRNS patients decreased significantly after RTX, as compared to before RTX (*P* = 0.001). The median height *z*-score at the time of RTX initiation was −0.8 and the median height *z*-score at the last follow-up visit was −0.7 (*P* = 0.81). The median BMI *z*-score decreased from 0.3 at the time RTX was initiated to −0.1 at the last follow-up visit (*P* = 0.11).

**Conclusion:** RTX has a more positive effect on disease outcome and growth in SSNS patients than in those with SRNS.

## Introduction

Idiopathic nephrotic syndrome (NS) is a major challenge in pediatric nephrology. The mainstay of NS treatment is steroid therapy. Nearly 80–90% of NS patients respond to steroid therapy and are considered to have steroid-sensitive NS (SSNS) (1). Approximately 60% of SSNS patients develop frequently relapsing NS (FRNS) or steroid-dependent NS (SDNS). In contrast, 10–20% of NS patients have steroid-resistant NS (SRNS) and do not respond to steroid therapy (2). Some SRNS patients can achieve partial or complete remission with calcineurin inhibitor (CNI) treatment (3). How best to treat NS patients that do not respond to steroids or CNIs remains a challenge. Moreover, patients with both FRNS/SDNS and SRNS usually require long-term steroid therapy, which is associated with the risk of serious side effects, including obesity, growth retardation, osteoporosis, glaucoma, cataracts, and hypertension (2).

Due to the problems associated with treating NS with steroids and CNIs, rituximab (RTX) has recently become an important alternative treatment option. RTX is an immunoglobulin IgG1-kappa type, mouse-human chimeric monoclonal antibody, with murine anti-CD20 variable sequence regions and human constant sequence regions. RTX binds specifically to the CD20 antigen, which is expressed on pre B-cells, and immature, mature, and memory B-cells, but not on plasma cells (4). Benz et al. (5) were the first to report the use of RTX in NS patients. Subsequently, additional case reports provided additional observational evidence of the positive effect of RTX on NS (6). Large series on the use of RTX reported that other immunosuppressive drugs could be withdrawn. However, the relapse rate is high when the CD19 and CD20 counts returned to normal (7). Reports of the efficacy of RTX for treating both SSNS and SRNS have been encouraging (8, 9).

The first RTX protocols for NS were based on lymphoma, with a once-weekly dose of 375 mg/m^2^ for 4 weeks. This protocol was modified in many institutions as 2 weekly doses of 750 mg/m^2^ plus maintenance dosing at regular intervals (4). In addition, some RTX researchers focused on RTX's effect on height and weight in children (2). Accordingly, the present study aimed to determine the long-term effects of RTX treatment on disease progression in children with SSNS and SRNS, and to analyze its effect on height and weight.

## Materials and Methods

### Patients and Definitions

Medical records of Caucasian SSNS and SRNS patients aged 1–20 years that were treated with RTX between 2009 and 2017 were retrospectively reviewed. Patients that were included in the study met the following criteria: 1. Onset of idiopathic NS at age >1 year; 2. Initiation of RTX treatment at age <19 years. Standard definitions were used for NS, remission, relapse, and steroid resistance (10, 11). Frequently relapsing NS was defined as ≥2 relapses within 6 months of achieving initial remission or ≥4 relapses in any 12-month period. SDNS was defined as 2 consecutive relapses while tapering the prednisolone dose or within 15 d of prednisolone discontinuation. SRNS was defined as lack of remission despite prednisolone 2 mg/kg/d (maximum: 60 mg/d) for 4 weeks, followed by 3 methylprednisolone pulses (12). Complete remission was defined as a urinary protein/creatinine ratio (Up/Uc) <0.2 mg/mg and partial remission was defined as a Up/Uc of 0.2–2 mg/mg and serum albumin >2.5 g/dL. All SRNS patients were screened for genetic mutations that cause steroid resistance.

The study protocol was approved by the Hacettepe University Ethics Committee (GO18/1136) and written informed consent was provided by the patients' parents and patients who were >10 years of age. The study was conducted in accordance with the World Medical Association Declaration of Helsinki.

### Immunosuppressive Therapies

Steroid therapy was initiated as 2 mg/kg/d (maximum: 60 mg/d) and continued for 4 weeks, followed by tapering as 1.5 mg/kg/d (maximum: 60 mg/d) q.o.d. for 4 weeks, 1 mg/kg/d q.o.d. for 4 weeks, 0.5 mg/kg/d q.o.d. for 4 weeks, and then 0.25 mg/kg/d on q.o.d. for 6–12 months (13). Relapse was treated with prednisolone 2 mg/kg/d (maximum: 60 mg/d) until remission (zero or trace proteinuria for 3 consecutive d), followed by prednisolone 2 mg/kg/d q.o.d. for 2 weeks, and then tapering (1 mg/kg/d q.o.d. for 2 weeks, 0.5 mg/kg/d q.o.d. for 2 weeks, and 0.25 mg/kg/d q.o.d. for 2 weeks and continued for 12 months). Cyclosporine 1–4 mg/kg/d b.i.d. was administered to maintain a through level of 50–100 ng/mL. Cumulative steroid and CNI dosages before and after RTX were also calculated. After obtaining parental/patient consent, patients were administered 2–4 weekly doses of RTX (375 mg/m^2^/dose).

Each patient's attending physician decided on the number of RTX doses for induction and maintenance. Premedication consisted of diphenhydramine, acetaminophen, and methylprednisolone (1 mg/kg, max. 60 mg) 30 min before RTX infusion. The standard methylprednisolone dose was also included in the calculation of cumulative steroid doses. Maintenance RTX treatment was given to some patients every 6–12 months, according to the clinical assessment made by their physician. Following RTX treatment, prophylactic trimethoprim-sulfamethoxazole t.i.w. was routinely administered. In order to prevent relapse, maintenance immunosuppressive therapy was continued, which could be stopped in some of the patients. CD19 and CD20 counts were not performed in all the patients on a regular basis.

### Growth Parameters

Height, weight, and BMI *z*-scores were assessed at the time RTX was initiated and at the last follow-up visit. Standard height, weight, and BMI scores were based on Turkish children growth curves. BMI was calculated as kg/height (m)^2^.

### Statistical Analysis

Descriptive statistical analysis was used to evaluate demographic and clinical data. Mean ± SD, median, and IQR (interquartile range) were calculated for numeric variables. Frequency tables were used to describe categorical data. The Mann-Whitney *U*-test or the independent *t*-test was used to compare 2 independent samples. Survival analysis was performed using Kaplan-Meier analysis with overall log-rank testing. All data were analyzed using IBM SPSS Statistics for Windows v.21 (IBM Corp., Armonk, NY).

## Results

Among the 41 pediatric NS patients included in the study, 21 (51.2%) had SSNS (20 SDNS and 1 FRNS) and 20 (48.8%) had SRNS. Mean duration of follow-up after RTX treatment was 2.3 ± 1.6 years. Additional patient clinical characteristics are given in the Table 1.

**Table 1:** Demographic and clinical characteristics of the SSNS and SRNS patients.

**Parameters**	**Total**	**SSNS**	**SRNS**	P^**^
Boys (*n*) (%)/Girls (*n*) (%)	22(53.7)/19 (46.3)	10 (47.6)/11 (52.4)	8 (40)/12 (60)	0.43
Age of diagnosis of NS^*^ (mean ± SD) (years)	5.8 ± 4.7	4.4 ± 3.6	7.3 ± 5.2	0.17
Age of first RTX^*^ treatment (mean ± SD) (years)	10.8 ± 5.1	10.8 ± 4.3	10.9 ± 5.6	0.62
Duration of follow-up after RTX^*^ treatment (mean ± SD) (years)	2.3 ± 1.6	2.5 ± 1.8	2.0 ± 1.4	0.44
Age at last visit (mean ± SD) (years)	13.0 ± 5.5	13.1 ± 5.6	12.9 ± 5.5	0.95
Number of the patients with renal biopsy before RTX^*^ (FSGS/MCD/MGN/C1qN^*^) (*n* = 36)	19/14/2/1	5/10/1/0	14/4/1/1	0.64
Duration of steroid treatment before RTX^*^ treatment (for SSNS; mean ± SD, for total and SRNS; median, IQR) (years)	4.3 (2.0–8.4)	6.4 ± 3.8	2.2 (1–5.8)	**0.028**
Duration of cyclosporine treatment before RTX^*^ treatment (for SSNS; mean ± SD, for total and SRNS; median, IQR) (years)	1.5 (0.8–6)	3.5 ± 2.7	0.8 (0.5–2.8)	**0.018**
Serum albumin level at the time of RTX^*^ (mean ± SD) (g/dl)	3.1 ± 0.9	3.7 ± 0.7	2.5 ± 0.8	**<0.001**
Urinary protein/creatinine ratio at the time of RTX^*^ (median) (IQR) (mg/mg)	5.6 (0.14–7.9)	0.14 (0.1–1.3)	6.8 (1.4–15.4)	**<0.001**
Steroid dosage before RTX^*^ (0–12 months) (median) (IQR) (mg/kg)	112.5 (57.6–176)	117.8 (75.6–152)	77 (46.7–240.5)	0.98
Steroid dosage after RTX^*^ (0–12 months) (median) (IQR) (mg/kg)	36.3 (21–62)	34 (20–48)	56.2 (21.8–95.5)	0.15
Steroid dosage before RTX^*^ (12–24 months) (median) (IQR) (mg/kg)	87.5 (53.5–220.5)	69.6 (51.4–133)	166 (56.3–572.3)	0.19
Steroid dosage after RTX^*^ (12–24 months) (median) (IQR) (mg/kg)	28.7 (7.5–74.8)	24.5 (7.5–74.8)	56.5 (9.4–83.5)	0.68
Cyclosporine dosage before RTX^*^ (0–12 months) (median) (IQR) (mg/kg)	831 (596.5–1057)	811.5 (594.8–937.5)	927 (496–1187.5)	0.34
Cyclosporine dosage after RTX^*^ (0–12 months) (median) (IQR) (mg/kg)	743 (316.8–909)	583.5 (305.8–903)	770.5 (336.8–925.4)	0.63
Cyclosporine dosage before RTX^*^ (12–24 months) (median) (IQR) (mg/kg)	750 (446–934.5)	782.5 (519–1057.3)	630 (397–929.5)	0.49
Cyclosporine dosage after RTX^*^ (12–24 months) (median) (IQR) (mg/kg)	436 (0–735)	725 (81.5–798)	405.5 (0–555.5)	0.32

* FSGS, focal segmental glomerulosclerosis; GN, glomerulonephritis; IQR, inter-quartile range; MCD, minimal change disease; MGN, membranous glomerulonephritis; NS, nephrotic syndrome; RTX, rituximab; SD, standard deviation.

P** is for comparison of related values of SSNS and SRNS. *Bold values indicate the statistically significant (<0.05) parameters*.

### SSNS Patients

In all, 21 (51.2%) of the NS patients had SSNS. All of these patients used steroid and CNI before RTX. Additional clinical features are given in the Table 1. The mean time from diagnosis of NS to the first RTX infusion was 6.4 ± 3.8 years. As induction treatment, RTX was given as 2, 3, and 4 doses in 4, 10, and 7 patients, respectively. Regular maintenance RTX treatment (every 6–12 months) was given to 10 patients (47.6%). Median number of cumulative RTX infusions was 4 (IQR; 3–6). Following RTX treatment, as maintenance treatment cyclosporine A was given to 10 of the patients and mycophenolate mofetil (MMF) was given to 2.

The median number of relapses during the first 2 years after RTX was 0 (IQR; 0–2), as compared to 4 (IQR; 2.5–6) during the 2 years before RTX (*P* = 0.001). In total, 8 (38%) of the SSNS patients relapsed after RTX. Among the relapsed patients, the mean time from RTX discontinuation to first relapse was 14.6 ± 11.7 months. In addition, among the relapsed patients RTX induction treatment was administered as 2 doses in 2 patients, 3 doses in 2 patients, and 4 doses in 4 patients. Kaplan-Meier analysis showed that age at diagnosis of NS, time from diagnosis of NS to RTX, age at initiation of RTX, renal histological diagnosis before RTX, and the number of RTX doses for both induction and maintenance were not associated with the first relapse after RTX treatment. The post-RTX relapse-free period did not differ significantly between the patients that received RTX induction treatment as 2, 3, and 4 doses (*p* = 0.48).

At the last follow-up visit 2 (9.5%) of the SSNS patients were in relapse, 3 (14.3%) in partial remission, and 16 (76.2%) in complete remission. At the last follow-up visit 6 of the patients were steroid free, 11 were still receiving steroid treatment of ≤0.5 mg/kg/d, and 4 were receiving steroid treatment at 0.5–1.1 mg/kg/d. At last follow-up visit, 11 of the SSNS patients were CNI free, 10 were still receiving CNI 2.4 ± 1.0 mg/kg/d, and 4 were both CNI free and steroid free.

**Figure 1:**
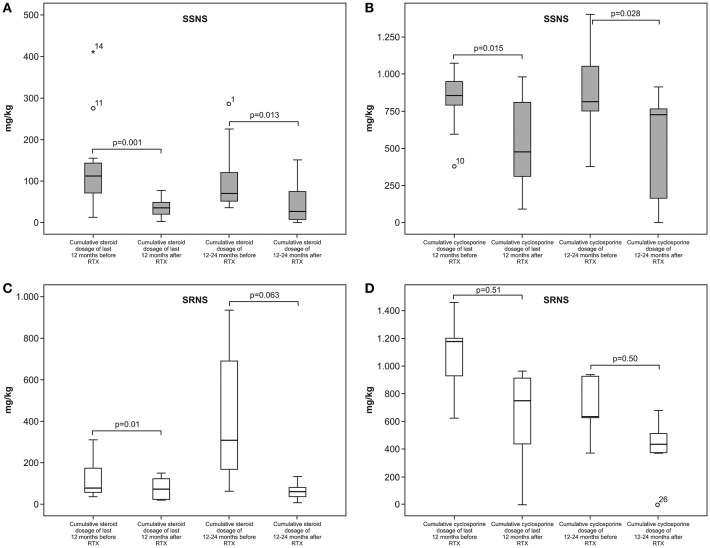
**(A,B)** cumulative steroid and cyclosporine doses in the SSNS patients 12 months before and 12 months after RTX treatment. **(C,D)** cumulative steroid and cyclosporine doses in the SRNS patients 12 months before and 12 months after RTX treatment.

Median cumulative steroid dose was 117.8 mg/kg (IQR; 75.6–152 mg/kg) for 0–12 months before RTX and 69.6 mg/kg (IQR; 51.4–133 mg/kg) for 12–24 months before RTX. At 0–12 months post RTX treatment the median cumulative steroid dose decreased to 34 mg/kg (IQR; 20–48 mg/kg; *P* = 0.001) and 24.5 mg/kg (7.5–74.8 mg/kg) 12–24 months post RTX (*P* = 0.013). The median cumulative cyclosporine dose was 811.5 mg/kg (IQR; 594.8–937.5 mg/kg) 0–12 months before RTX and 782.5 mg/kg (IQR; 519–1057.3 mg/kg) 12–24 months before RTX. At 0–12 months post RTX the median cumulative cyclosporine dose decreased to 583.5 mg/kg (IQR; 305.8–903 mg/kg) mg/kg (*P* = 0.015) and 725 mg/kg (IQR; 81.5–798 mg/kg) 12–24 months post RTX (*P* = 0.028; Figures 1A,B). Mean GFR at initiation of RTX and at the last follow-up visit was 202.1 ± 55.8 mL/min/1.73 m^2^ and 176.9 ± 50.1 mL/min/1.73 m^2^, respectively (*P* = 0.14).

At the initiation of RTX 18 (85.7%) of the SSNS patients had a height *z*-score <0 and their mean time from NS diagnosis to initiation of RTX was 6.6 ± 4.1 years. Among the SSNS patients, 3 (14.3%) had a height *z*-score >0 at initiation of RTX and their median time from NS diagnosis to initiation of RTX was 5.5 ± 1.4 years (*P* = 0.60). Steroid treatment duration of the patients with a height *z*-score <0 (6.8 ± 4.0 years) and >0 (4.0 ± 1.4 years) at the time of RTX was not statistically different (*P* = 0.35). Similarly, mean duration of cyclosporine treatment did not differ significantly according to height *z*-score (*z*-score <0: 3.7 ± 2.8 years; *z*-score >0: 2.0 ± 2.0 years; *P* = 0.51). In total, the height *z*-score in 11 (52.3%) SSNS patients improved after RTX treatment; the median height *z*-score at initiation of RTX was −1.2 [IQR; (−2.5)–(−0.3)], vs. −0.6 [IQR; (−1.9)–(−0.1)] at the last follow-up visit (*P* = 0.044). At the last follow-up visit the BMI *z*-score in 15 (71.4%) SSNS patients was lower than at initiation of RTX; the median BMI *z*-score decreased from 1.6 (IQR; 0.9–3.0) at the time of initiation of RTX treatment to 1.1 (IQR; [(−0.7)−2.5] at the last follow-up visit (*P* = 0.007).

### SRNS Patients

In total, 20 of the NS patients (48.8%) had SRNS, all of which were also resistant to CNIs. Mean duration of follow-up after RTX was 2.0 ± 1.4 years. Additional clinical features are given in the Table 1. RTX induction treatment was administered as 2 doses in 2 patients, 3 doses in 4 patients, and 4 doses in 14 patients. Among the SRNS patients, 14 (70%) received maintenance RTX every 6–12 months. Median number of cumulative RTX infusions was 6 (IQR; 4–7). Rituximab treatment was discontinued in 5 SRNS patients that progressed to chronic renal failure (<90 ml/min/1.73 m^2^).

At the last follow-up visit 4 (20%) of the SRNS patients had complete remission and 4 (20%) had partial remission, whereas 6 patients still had active disease with normal GFR (≥90 ml/min/1.73 m^2^) and 6 others had a GFR <90 mL/min/1.73 m^2^. Steroids were discontinued in 2 SRNS patients with complete remission and partial remission, and cyclosporine was withdrawn in 4 patients with partial remission. The 1-year cumulative steroid dose decreased significantly after RTX, as compared to before RTX (0–12 months). In contrast, the 1-year cumulative cyclosporine dose before and after RTX did not differ significantly (Figures 1C,D).

The median cumulative steroid dose 0–12 months before RTX was 77 mg/kg (IQR; 46.7–240.5 mg/kg), and 166 mg/kg (IQR: 56.3–572.3 mg/kg) for 12–24 months before RTX. The median cumulative steroid dose 0–12 months post RTX decreased to 56.2 mg/kg (IQR; 21.8–95.5 mg/kg; *P* = 0.01) and 56.5 mg/kg (IQR: 9.4–83.5 mg/kg) 12–24 months post RTX (*P* = 0.063). The median cumulative cyclosporine dose was 927 mg/kg (IQR; 496–1187.5 mg/kg) 0–12 months before RTX, and 630 mg/kg (IQR; 397–929.5 mg/kg) 12–24 months before RTX. The median cumulative cyclosporine dose 0–12 months post RTX decreased to 770.5 mg/kg (IQR; 336.8–925.4 mg/kg; *P* = 0.51), as compared to 405.5 mg/kg (IQR; 0–555.5 mg/kg) 12–24 months post RTX (*P* = 0.80; Table 1). Mean GFR at initiation of RTX and at the last follow-up visit was 214.6±181.6 mL/min/1.73 m^2^ and 128.5±63.2 mL/min/1.73 m^2^, respectively (*P* = 0.28).

At the initiation of RTX 15 of the SRNS patients had a height *z*-score <0 and their median time from NS diagnosis to RTX initiation was 3.9 ± 3.9 years. Five patients had a height *z*-score >0 and their median time from NS diagnosis to RTX initiation was 2.8 ± 2.5 years (*P* = 0.80). The duration of steroid treatment did not differ significantly between the SRNS patients with a height *z*-score <0 (7.2 ± 10.4 years) and >0 zero (2.0 ± 1.5 years) at RTX initiation (*P* = 0.23). Similarly, the duration of cyclosporine treatment in the patients with a height *z*-score <0 (2.4 ± 3.7 years) and >0 (1.7 ± 1.3 years) at initiation of RTX did not differ significantly (*P* = 0.74). The height *z*-score improved in 8 of the SRNS patients after RTX treatment; the median height *z*-score at initiation of RTX was −0.8 [IQR; (−2.2)–(0.03)], vs. −0.7 [IQR; (−2.1)−0.4] at the last follow-up visit (*p* = 0.81). At the last follow-up visit the BMI *z*-score in 13 (65%) SRNS patients was lower than at initiation of RTX; the median BMI *z*-score decreased from 0.3 [IQR; (−0.8)−1.0] at initiation of RTX to −0.1 [IQR;(−1.0)−0.8] at the last follow-up visit (*p* = 0.11).

### Adverse Effects

Only 1 SSNS patient and 2 SRNS patients had mild RTX infusion-related adverse reactions (tingling of the throat). In all patients, the rate of RTX infusion was decreased and these adverse effects did not persist. Additionally, they did not re-occur in following RTX doses. All patients continued RTX doses. None of the patients had serious infections, such as peritonitis, cellulitis, and thrombosis, or other adverse events.

## Discussion

The present study aimed to determine the effects of RTX on disease outcome and growth parameters in pediatric SSNS and SRNS patients. Fornoni et al. (14) observed that RTX can bind directly to the sphingomyelin phosphodiesterase acid-like 3b protein (SMPDL3b), which is a component of phospholipid membranes that prevents SMPDL3b down-regulation in podocytes. RTX's relevance to NS was first reported in 2004 (5); subsequently, numerous studies on RTX and NS were performed: however, there remains a lack of consensus regarding the use of RTX for treating NS, including the optimal regimen for initial treatment (dose and number of doses). Kamei et al. (15) reported that Japanese patients with a single dose of RTX had a higher rate (75%) of relapse than those given multiple doses. The researchers concluded that the ability of a single RTX dose to prevent relapses was transient and that most relapses occur in association with recovery of B-cells. Kemper et al. (7) studied 37 SDNS patients and reported that among the 21 that received 1 dose of RTX, 64.8% relapsed. The researchers also noted that the time to first relapse was shorter in the patients that received 1 or 2 doses of RTX, as compared to those that received 3 or 4 doses. Lastly, they reported that the number of RTX induction doses did not affect long-term prognosis.

Fujinaga et al. (16) studied the predictors of relapse and long-term outcome in 43 SDNS patients given a single dose of RTX as induction treatment. In all, 91% of their patients relapsed. In addition, the relapse-free survival rate in the patients aged <12.5 years at the time of initial RTX treatment was shorter than in those aged ≥12.5 years. Furthermore, age at RTX initiation and the duration of relapse-free periods was positively correlated. The researchers also noted that the early relapse rate after RTX was significantly higher in the patients with shorter CD19 depletion. Tellier et al. (17) studied the long-term outcome in 18 SDNS patients with a median duration of follow-up of 3.2 years. They observed a relapse rate of 56% during a 2-year period following RTX treatment. In addition, 44.5% of the patients were free of therapy at the last follow-up visit. In the present study 38% of the SSNS patients relapsed following RTX treatment, but age and the number of RTX doses administered for both induction and maintenance were not associated with the first post-RTX relapse. Furthermore, in the present study 20% of SSNS patients were free of oral drug therapy.

Findings regarding the efficacy of maintenance immunosuppressive drug treatment following RTX are inconsistent. Ito et al. (18) reported that maintenance therapy with MMF is effective in SDNS patients, whereas Fujinaga et al. (19) compared MMF and cyclosporine after 1 dose of RTX in SDNS patients, reporting that treatment failure occurred more frequently in those given MMF and that the sustained remission rate was higher in the cyclosporine group. In the present study most of the NS patients received cyclosporine as maintenance therapy and the type of maintenance therapy did not affect the time to first relapse after RTX treatment.

Sinha et al. (8) published one of the largest studies on RTX in NS (SSNS and SRNS) patients. They included 101 SDNS patients, of which 82 received 2 doses of RTX initially. The authors found that both cumulative steroid dosage and number of relapses were lower at in time periods 6 and 12 months after RTX compared to 6 and 12 months prior RTX. Similarly, in the present study the cumulative steroid dose 12 months after RTX was lower than 12 months before RTX. Moreover, the present study also calculated the cyclosporine dose before and after RTX, observing that the cumulative cyclosporine dose was lower after RTX than before RTX. More recently, Ravani et al. (20) performed a multicenter, open label, non-inferiority trial with pediatric SDNS patients that had developed SDNS during the previous the 6–12 months and were maintained in remission with high-dose prednisone. Their patients were divided into 2 groups: prednisone (control) and single-dose RTX (intervention). They concluded that RTX was not inferior to steroids for the treatment of SDNS.

The rate of response to RTX in SRNS patients is lower than in SSNS patients, even when SSNS patients receive RTX during periods of relapse. The first report of RTX treatment in SRNS patients included 5 Indian children treated with RTX, of which 3 achieved complete remission and 2 achieved partial remission (21). Prytula et al. (22) studied 27 SRNS patients with different RTX regimes. In this cohort, 17 patients received two doses of 750 mg/m^2^ every 14 days and 6 patients received single dose of RTX (375 mg/m^2^). The rest of the patients received other treatment regimes of RTX. After these RTX doses, 6 patients (22%) achieved full remission and 6 patients (22%) had proteinuria with serum albumin >30 g/L. Sinha et al. (8) reported a series of 58 NS patients that were steroid and calcineurin resistant. Most of the patients (*n* = 39) received 4 doses of RTX, and complete remission and partial remission were observed in 12.1 and 17.2% of the patients, respectively. Fujinaga et al. (23) studied the long-term outcome of early RTX treatment in 6 NS patients that were both cyclosporine and steroid resistant. Following RTX treatment, and then intravenous pulse methylprednisolone and/or high-dose prednisolone, all patients achieved complete remission.

The findings above, in whole, show that the response to RTX varies geographically. The present study's findings fall between those of the 2 geographic regions described above. Complete or partial remission was observed in 40% of the present study's patients, respectively. Some of the SSNS and SRNS patients in the present study received periodic RTX dose, as reported earlier (24). Based on the present findings, we think that such dosing regimens might have a positive effect on remission.

The effect of RTX on growth in children is another contentious issue. Kamei et al. (25) measured the height of 21 SSNS patients aged ≤10 years at initiation of RTX and at the last follow-up visit, reporting that the height *z*-score did not differ significantly between the 2 time points. Sato et al. (2) investigated the effect of RTX on height and weight in 13 SDNS patients, and observed that the height *z*-score for all 13 patients was higher at the last follow-up visit and the height *z*-score of 10 of the 13 patients increased significantly following RTX treatment. Lastly, they noted that the obesity index in 12 of the 13 patients improved significantly following RTX treatment. More recently, Basu et al. (26) compared the efficacy of RTX and tacrolimus in SDNS patients in a randomized clinical trial. They reported a higher 12-month absolute change in the height *z*-score in the RTX arm, as compared to the tacrolimus arm. In the present study the height *z*-score in 50% of the patients improved following RTX treatment and the median height *z*-score at the last follow-up visit was higher than at the time of initiation of RTX treatment. RTX had a similarly positive effect on BMI in the present study's SSNS and SRNS patients. These positive effects of RTX on growth were less prominent in SRNS patients than in the SSNS patients; only 40% of the SRNS had an improved height *z*-score after RTX treatment, and the median height *z*-score among the SRNS patients at the last follow-up visit and at the time of initiation of RTX did not differ significantly. These findings might have been due to the shorter duration of steroid treatment before RTX in SRNS patients than in the SSNS patients.

In conclusion, the positive effects of RTX on disease progression and growth (height and BMI) are more prominent in SSNS patients than in SRNS patients. RTX causes decreased number of relapses, cumulative steroid, and calcineurin dosages.

## Data Availability

The raw data supporting the conclusions of this manuscript will be made available by the authors, without undue reservation, to any qualified researcher.

## Author Contributions

RT, BG, FO, and AD: research formulation and study design. KÇ, Mİ, and BG: data acquisition. RT, BG, and MH: data analysis/interpretation. MH and BG: statistical analysis. RT: supervision/mentorship. Each of the authors contributed important intellectual content during manuscript drafting and/or revision, and approved the final version. Furthermore, they all accept responsibility for the overall work, including the accuracy and integrity of all portions of the work.

### Conflict of Interest Statement

The authors declare that the research was conducted in the absence of any commercial or financial relationships that could be construed as a potential conflict of interest.
